# Supplementary material for: Backscattering in topological edge states despite time-reversal symmetry
Source: Nat Commun. 2025 Sep 2;16:8209. doi: 10.1038/s41467-025-63572-2 (PMC12405585; doi:10.1038/s41467-025-63572-2)
Supplement: Supplementary file 1 — Supplementary Information [file 41467_2025_63572_MOESM1_ESM.pdf]

Supplemental Information:
Backscattering in Topological Edge States Despite Time-Reversal Symmetry

Jonas Erhardt,[1,2] Mattia Iannetti,[3] Fernando Dominguez,[2,4] Ewelina M. Hankiewicz,[2,4] Björn Trauzettel,[2,4] Gianni Profeta,[3,5] Domenico Di Sante,[6] Giorgio Sangiovanni,[2,4] Simon Moser,[1,2] and Ralph Claessen[1,2,*]

[1] *Physikalisches Institut, Universität Würzburg, D-97074 Würzburg, Germany*
[2] *Würzburg-Dresden Cluster of Excellence ct.qmat, Universität Würzburg, D-97074 Würzburg, Germany*
[3] *Department of Physical and Chemical Sciences, University of L'Aquila, Via Vetoio, 67100, L'Aquila, Italy*
[4] *Institut für Theoretische Physik und Astrophysik, Universität Würzburg, D-97074 Würzburg, Germany*
[5] *SPIN-CNR, University of L'Aquila, Via Vetoio 10, 67100, L'Aquila, Italy*
[6] *Department of Physics and Astronomy, University of Bologna, Bologna, Italy*

(Dated: August 18, 2025)

CONTENTS

Supplementary Discussion I 2

Supplementary Discussion II 4

Supplementary Discussion III 6

Supplementary Discussion IV 10

Supplementary Discussion V 12

Supplementary Discussion VI 14

Supplementary Discussion VII 16

References 18

* e-mail: claessen@physik.uni-wuerzburg.de

SUPPLEMENTARY DISCUSSION I

Both the indenene bulk and its flat edge states are highly sensitive to the local potential induced by the carbon atom in the topmost SiC substrate layer. According to the absence and presence of this carbon atom *between* the indenene atoms, this necessitates a distinction between positions A and B, respectively, within the indenene bulk unit cell (inset in Fig. S1**a**), as well as between flat edge terminations by position A or B (Fig. S1**a**). The unambiguous identification of the flat edge termination is thus essential for investigating their electronic properties.

However, from a macroscopic viewpoint, the lattice orientation and thus edge termination is not fully determined due to the hexagonal polytypism of the 4H-SiC(0001) substrate. The latter causes the surface lattice orientation to change by 180° when crossing a half (0.5 nm) SiC steps from one indenene covered SiC terrace to another, see Fig. S1e. Without additional information, this makes it impossible to identify the flat edge orientation neither in overviewscans at the 100 nm scale (Fig. S1b) nor in atomically resolved scans of the clean indenene surface (Fig. S1a). To resolve this issue, we leverage the fact that nitrogen dopants in the SiC substrate substitute exclusively for carbon in the SiC lattice [1, 2], thereby providing a clear identifier for the carbon position in the first and second SiC layer and thus pinpointing site B and A of the indenene lattice, respectively. Consequently, for every edge investigated in this study, we first calibrate the associated SiC terrace based on the carbon position within the indenene bulk unit cell.

In general, we identify three types of three-fold symmetric defects (Fig. S1c, d), that correspond to single-atoms and consistently appear in the same orientation on a clean indenene-covered SiC terrace. While defect D3 (Fig. S1d_3) appears relatively pronounced in STM images and conceals the indenene lattice entirely, defects D1 (Fig. S1d_1) and D2 (Fig. S1d_2) appear less protruding and do not fully mask the indenene lattice. Therefore, in analogy to Ref. 3, we attribute defects D1 and D2 to substitutional nitrogen doping atoms allocated in the first (D1) and second (D2) SiC layers, as schematically illustrated in Fig. S1e, while D3 is likely a surplus (indium) atom that has adsorbed onto the indenene lattice. It is noteworthy that the orientation of adatom D3 is also fixed for each indenene terrace, indicating a favored adsorption position of surplus indium.

Focusing on the lattice position of defect D1 and D2 (Fig. S1d_1 and d_2), we observe that their defect centers occupy different sites within the indenene unit cell. This is consistent with the carbon positions of the two topmost SiC layers shown in the top view of Fig. S1e. Furthermore, the nitrogen concentration in a bulk layer of our SiC wafers is approximately $3/(20\ \text{nm})^2$ [4], which aligns well with the number of defects (D1 and D2) in Fig.1c and larger scans in Fig.1f. Discrepancies might be caused by nitrogen diffusion during the H-etching procedure at 1250°C conducted during substrate preparation.

To test the robustness of this method for determining the terrace orientation, we investigate adjacent indenene-covered SiC terraces separated by a half 4H-SiC(0001) unit cell. As described above, the 180° rotation inherent to the hexagonal SiC lattice must also affect the orientation of the N-dopant, see Fig. S1e. Indeed, we observe that defect D1 and D2 change their orientation by 180° between the upper and lower terrace shown in Fig. S1f_1 and f_2. This clearly matches the expected behavior of carbon substituting nitrogen in the SiC lattice (Fig. S1f_1 and f_2), allowing us to immediately identify the upper and lower indenene flat edges as B-terminated.

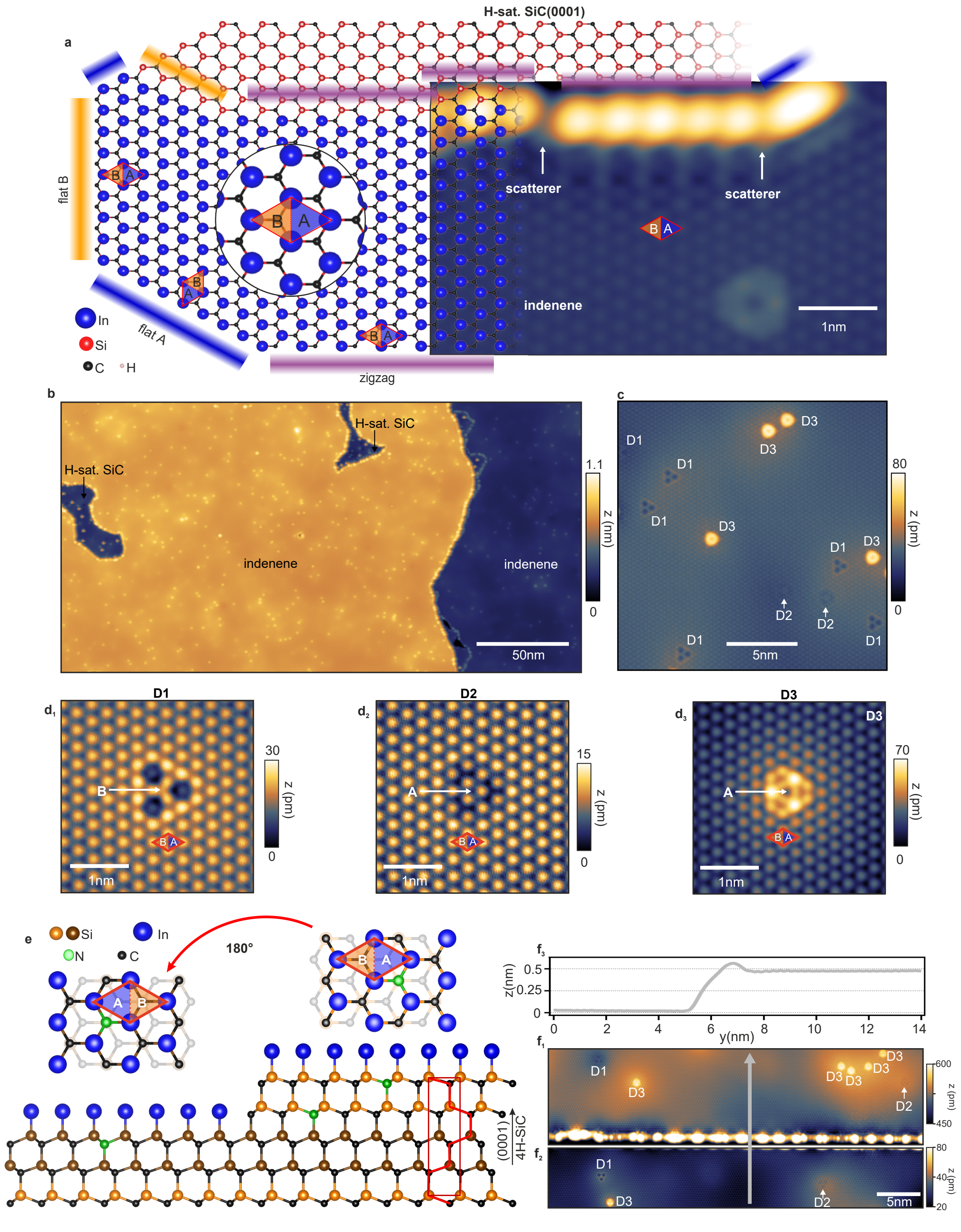

Figure S 1. **a** Ball and stick model (left) of indenene on SiC(0001) forming the three edge types (zigzag, flat A, flat B) to H-saturated SiC. An inset highlights position A and B of the indenene unit cell, revealing the presence of the carbon atom of the topmost SiC layer at position B. STM topography (right) of an indenene edge consisting of zigzag and flat edge A segments interrupted by kinks that act as scattering centers ($I_{\mathrm{T}} = 300\,\mathrm{pA}$, $V_{\mathrm{bias}} = 0.95\,\mathrm{V}$). **b** STM overviewscan showing holes with H-sat. SiC in the otherwise closed indenene film ($I_{\mathrm{T}} = 10\,\mathrm{pA}$, $V_{\mathrm{bias}} = 3\,\mathrm{V}$) as well as a 0.5 nm SiC terrace step to a lower indenene covered SiC terrace. **c** Atomically resolved STM scan of indenene showing three types of defect labeled D1, D2 and D3. Tunneling parameters were $I_{\mathrm{T}} = 500\,\mathrm{pA}$, $V_{\mathrm{bias}} = 0.8\,\mathrm{V}$. **d** Close-up STM scans of defect D1 ($\mathbf{d}_1$), D2 ($\mathbf{d}_2$) and D3 ($\mathbf{d}_3$) taken at scan parameters optimized for best defect imaging; $\mathbf{d}_1$: $I_{\mathrm{T}} = 500\,\mathrm{pA}$, $V_{\mathrm{bias}} = 0.8\,\mathrm{V}$, $\mathbf{d}_2$: $I_{\mathrm{T}} = 500\,\mathrm{pA}$, $V_{\mathrm{bias}} = 1\,\mathrm{V}$, $\mathbf{d}_3$: $I_{\mathrm{T}} = 500\,\mathrm{pA}$, $V_{\mathrm{bias}} = 0.5\,\mathrm{V}$. The overlaid indenene unit cell indicates that D1 and D3 are centered at lattice site T_4 (B) and D2 at H_3 (A). **e** Top and side view ball and stick model of an indenene-covered 4H-SiC(0001) step structure, emphasizing a 180° rotation in the 4H-SiC stacking order at every half unit cell (red arrow). The top view shows a nitrogen atom replacing carbon in the first SiC layer, likely corresponding to D1 and indicating site B. Similarly, carbon substitution by nitrogen in the second SiC layer indicates site A within the indenene unit cell. **f** Atomically resolved STM scan of an indenene-covered 4H-SiC(0001) step configuration as shown in **b** taken at $I_{\mathrm{T}} = 50\,\mathrm{pA}$, $V_{\mathrm{bias}} = 0.6\,\mathrm{V}$. For clarity, the scan is divided into the upper ($\mathbf{f}_1$) and lower terrace ($\mathbf{f}_2$). The inset arrow highlights the height profile in $\mathbf{f}_3$, demonstrating that the two terraces are separated by a half (0.5 nm) 4H-SiC(0001) step.

SUPPLEMENTARY DISCUSSION II

In this section, we discuss the bulk decay length of indenene flat edge states at the example of edge segment 2 illustrated in Fig. 3 of the main text.

Fig. S2a presents a corresponding extended topography scan of Fig. 3a (main text), where we recorded line $\mathrm{d}I/\mathrm{d}V(E,x)$ spectroscopy from segment 2 into the indenene bulk, as indicated by a red arrow. We note that in this case the indenene band filling is in the degenerate doping regime, *i.e.*, the conduction band is partially occupied, as schematically indicated by the Fermi level position E_F in the inset of Fig. S2a. This band filling is directly evident in the bulk $\mathrm{d}I/\mathrm{d}V(E,x)$ spectrum (overlaid blue in Fig. S2b) that was taken far from the edge, where we identify the onsets of valence and conduction bands in the spectrum. The spectrum further reveals a zero bias anomaly (ZBA) at 0 V and the indenene bulk band gap, whose $\mathrm{d}I/\mathrm{d}V$ signal centered around -0.17 V does not drop entirely to zero, similar to what was observed in earlier work [5]. The non-vanishing $\mathrm{d}I/\mathrm{d}V$-signal in the bulk gap is not related to the edge vicinity nor to actual local density of states, but instead, it is attributed to the degenerate doping regime that influences the tunneling matrix element. For a detailed description of this artifact in STS measurements we refer to the Supplementary Information of Ref. 5.

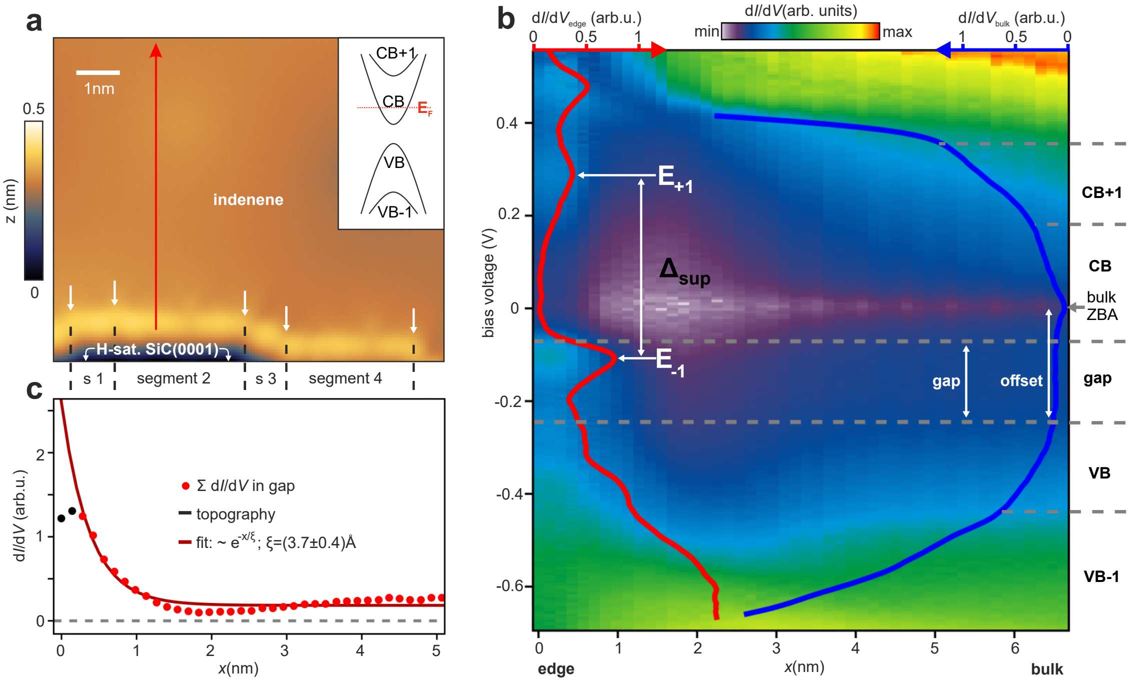

Figure S 2. **a** Topography STM scan of the indenene flat edge A to H-saturated SiC(0001) also shown in Fig. 3**a** of the main text. A red arrow indicates the path along which the STS line scan shown in **b** was recorded, while white arrows indicate edge registry shifts that separate straight edge segments. The inset illustrates the band filling with the Fermi level E_F situated in the conduction band (CB) shifting bulk band gap and valence bands (VB) to negative bias voltage in STS. **b** Line $\mathrm{d}I/\mathrm{d}V(E,x)$ scan from edge segment 2 into the indenene bulk recorded with tunneling setpoint $I_\mathrm{T} = 500\,\mathrm{pA}$, $V_\mathrm{bias} = -0.7\,\mathrm{V}$. Bulk zero bias anomaly (ZBA) [5], CB and VB onsets – the latter taken as zero energy for comparison to theory – as well as bulk gap bulk are indicated in the blue $\mathrm{d}I/\mathrm{d}V(E)$ spectrum taken 6 nm away from the edge. On the left side of the plot, an edge spectrum (red) is overlaid, highlighting the QPI related energy level E_{-1} and E_{+1} separated by Δ_sup. **c** The edge $\mathrm{d}I/\mathrm{d}V(E,x)$ signal exhibits exponential decay with a decay constant of $\xi = (3.7 \pm 0.4)$Å, exponentially fitted to the gap-integrated $\mathrm{d}I/\mathrm{d}V$ signal (red data points).

Approaching the edge, the $\mathrm{d}I/\mathrm{d}V(E,x)$ signal is first slightly reduced, potentially related to band bending in the vicinity of the edge, before a distinct state fills up and exceeds the bulk band gap. We identify this edge state as the q related E_{-1} energy level of segment 2, as also highlighted in the overlaid spectrum (red) that was taken directly at the edge. Comparing the energy position of the E_{-1} level and the bulk band gap demonstrates that the $n = 3$ Kramers pair situation is indeed present within the entire gap, consistent with our tight binding analysis. For better comparison between the energy level E_i and the tight-binding-derived momentum JDOS, the position of the valence band maximum (VBM) is used as a common energy reference (see Fig. 2**b**). Its energy position is determined by a linear fit to the valence band $\mathrm{d}I/\mathrm{d}V$ signal and the in-gap signal, with their intersection marking the valence band

maximum. Possible band bending effects are accounted for in the error of the offset value. We find the flat edge A in-gap differential conductance exponentially localized within $\xi = (3.7 \pm 0.4)$ Å (Fig. S2c), which agrees well with the edge localization determined from charge neutral indenene at zigzag edges (Fig. 2e in the main text). The error is estimated based on multiple exponential fits with different integration limits of the in-gap $\mathrm{d}I/\mathrm{d}V(E,x)$ signal.

SUPPLEMENTARY DISCUSSION III

Tight-binding model

The theoretical characterization of the edge states is performed using a 6 nearest-neighbor (NN) tight-binding (TB) model based on a Slater & Koster (SK) parametrization of hopping terms, similar to what is presented in Refs. 5–7. The Hamiltonian of the system is:

$$\hat{H} = \hat{H}_0 + \hat{H}_{HSB} + \hat{H}_{ISB} + \hat{H}_{SOC}, \tag{1}$$

where $\hat{H}_0$ is the free-standing indenene Hamiltonian, $\hat{H}_{HSB}$ and $\hat{H}_{ISB}$ are the out-of-plane and in-plane inversion symmetry-breaking parts of the Hamiltonian, and $\hat{H}_{SOC}$ represents the on-site spin-orbit coupling (SOC) interaction. $\hat{H}_0$ accounts for the hopping amplitudes of the indium p_x, p_y, and p_z orbitals up to the 6th NN. Explicitly:

$$\langle p_i(0)|\hat{H}_0|p_i(\boldsymbol{R})\rangle = n_i^2 V_i^\sigma + \left(1 - n_i^2\right) V_i^\pi, \tag{2}$$

$$\langle p_i(0)|\hat{H}_0|p_j(\boldsymbol{R})\rangle = -n_i n_j \left(V_{ij}^\pi - V_{ij}^\sigma\right), \tag{3}$$

where $i = x, y, z$, n_i is the in-plane orientation ($n_x = \cos\theta \sin\phi$, $n_y = \sin\theta \sin\phi$, $n_z = \cos\phi$) with θ and ϕ being the azimuthal and polar angles, respectively. $\boldsymbol{R}$ is the NN real-space vectorial distance, while $V_{xx}^\sigma = V_{yy}^\sigma = V_{xy}^\sigma = V^\sigma$, $V_{xx}^\pi = V_{yy}^\pi = V_{xy}^\pi = V^\pi$, and V_z^π are the non-zero SK integrals.

The $\hat{H}_{HSB}$ out-of-plane inversion symmetry-breaking potential arises due to the presence of the substrate. It is modeled by coupling of the p_z and $p_{x/y}$ orbitals, such that:

$$\langle p_i(0)|\hat{H}_{HSB}|p_z(\boldsymbol{R})\rangle = -n_i \lambda_{HSB}, \tag{4}$$

$$\langle p_z(0)|\hat{H}_{HSB}|p_i(\boldsymbol{R})\rangle \; s = n_i \lambda_{HSB}, \tag{5}$$

where $i = x, y$ and λ_{HSB} is the strength of the interaction.

The in-plane inversion symmetry Hamiltonian $\hat{H}_{ISB}$ is written in terms of allowed hoppings of the indium p orbitals to an auxiliary dispersionless s-orbital centered at the B site of the unit cell (UC), as depicted in Fig. S3. Following the SK parametrization, we get:

$$\langle s(0)|\hat{H}_{ISB}|p_i(\boldsymbol{R})\rangle = n_i V_{sp}^\sigma = V_{sp}^{i\sigma}. \tag{6}$$

With $V_{sp}^{z\sigma} = n_z V_{sp}^\sigma$ and $V_{sp}^{x/y\sigma} = n_{x/y} V_{sp}^\sigma$. This procedure explicitly considers the inversion symmetry breaking without the need of a down-folding process as in Ref. 7. The inversion symmetry breaking potential is then related to V_{sp}^σ as $\lambda_{ISB} \simeq -\frac{9}{4}\frac{V_{sp}^{\sigma\,2}}{E_s}$, given by the splitting at K/K' valleys of the Dirac cones, with E_s being the onsite energy of the auxiliary s-orbital. Finally, the SOC Hamiltonian $\hat{H}_{SOC}$ is written as an on-site term acting on indium p orbitals:

$$\hat{H}_{SOC} = \frac{\lambda_{SOC}}{2} \begin{pmatrix} 0 & -i\sigma_z & i\sigma_y \\ i\sigma_z & 0 & -i\sigma_x \\ -i\sigma_y & i\sigma_x & 0 \end{pmatrix}. \tag{7}$$

The construction and diagonalization of the Hamiltonian for the two edge terminations are performed using the Python package Pybinding [8]. The band structure, projections and momentum JDOS $\Delta\rho(\boldsymbol{q}, \omega)$ calculations are generated as the result of a post-processing Python library. The TB parameters are fitted to first-principles calculations based on the energy band structure of the 2D bulk system and reported in Table S1. Flat and zigzag edges are generated by constructing supercells on the order of ~ 400 atoms in the geometry depicted in Fig. S3. Based on density functional theory slab calculations (PBE), a constant edge potential is considered in TB slab calculations of flat edge terminations. Results are shown in Fig. S4 indicating spin and orbital character of the termination dependent edge bands.

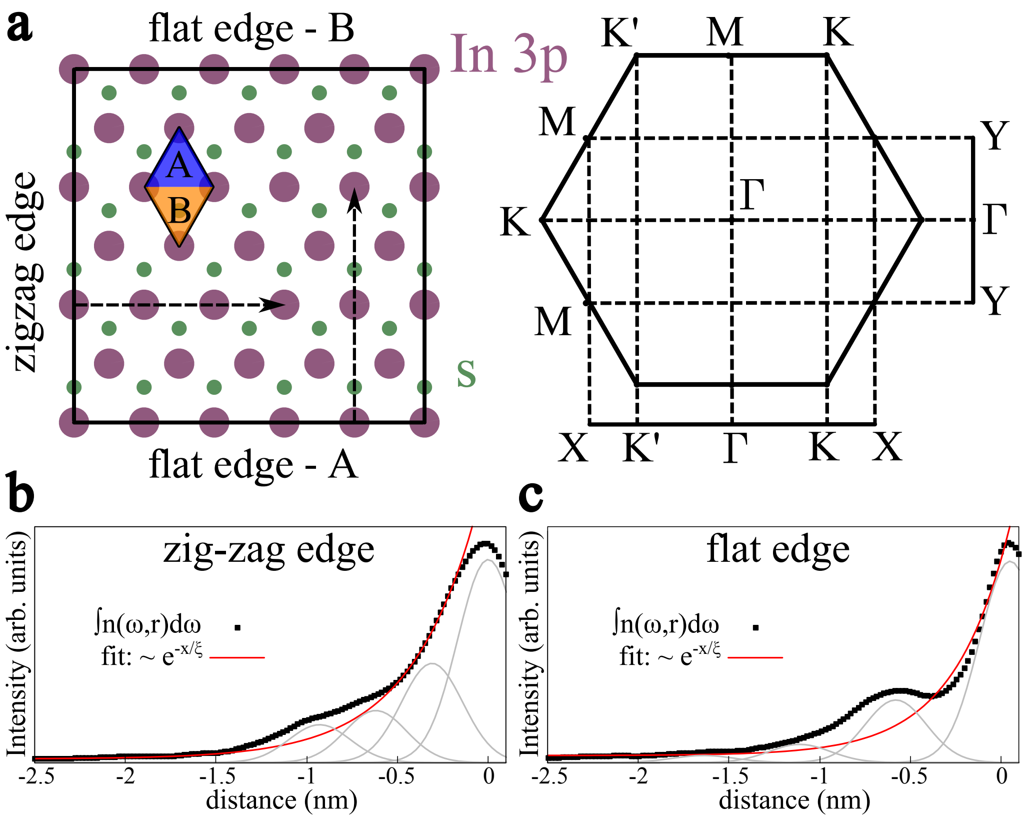

Figure S 3. **a** Sketch of the tight-binding model structure used, with the corresponding reciprocal space Brillouin zones projected onto the edges cut. **b**,**c** Calculated decay constants for the zigzag ($\xi = (3.7\pm0.3)$Å) and flat edge A ($\xi = (3.1\pm0.3)$Å) termination respectively. Gray lines in **b** and **c** are Gaussian functions centered at indium atom positions (see dashed line arrows in **a**).

V^{σ}	0.9267	-0.0104	-0.1422	0.1345	-0.0102	-0.0646
V^{π}	-0.2994	-0.0629	-0.0051	-0.0106	0.0809	-0.1036
V^{π}_{z}	-0.2271	-0.0926	-0.2165	0.0907	-0.0709	0.0766
E_z	-0.8815					
E_F	-1.7796					
E_s	3.5000					
$V^{z\sigma}_{sp}$	0.0997					
λ_{ISB}	0.2399					
λ_{SOC}	0.3005					
λ_{HSB}	0.5649					

Table S 1. Tight-binding parameters used in slab calculations. All values are expressed in eV.

T-matrix formalism

The theoretical QPI calculations are obtained using the T-matrix formalism as described in Refs. 9 and 10, where the momentum JDOS $\Delta\rho(\boldsymbol{q},\omega)$ is defined as:

$$\Delta\rho\left(\boldsymbol{q},\omega\right) = \sum_{\boldsymbol{k}}\sum_{n,m}\left|M_{n\boldsymbol{k},m\boldsymbol{k}+\boldsymbol{q}}\right|^{2} A_{n\boldsymbol{k}}\left(\omega\right) A_{m\boldsymbol{k}+\boldsymbol{q}}\left(\omega\right), \tag{8}$$

where $M_{n\boldsymbol{k},m\boldsymbol{k}'} = \langle \boldsymbol{k}n|V|\boldsymbol{k}'m\rangle$ is the scattering matrix element of a non-magnetic impurity potential $V = V_0\sigma_0$, with σ_0 being the identity matrix, and $A_{n\boldsymbol{k}}\left(\omega\right) = \frac{W_{n\boldsymbol{k}}}{\omega-\epsilon_{n\boldsymbol{k}}+i0^{+}}$ is the spectral function of the n-th band with momentum $\boldsymbol{k}$ at energy ω and $W_{n\boldsymbol{k}}$ is the unfolding spectral weight defined as in Ref. 11. To reproduce the one-dimensional nature of the measurements done along the edge states, only the spectral weight coming from edge atoms is considered in the $W_{n\boldsymbol{k}}$ calculation.

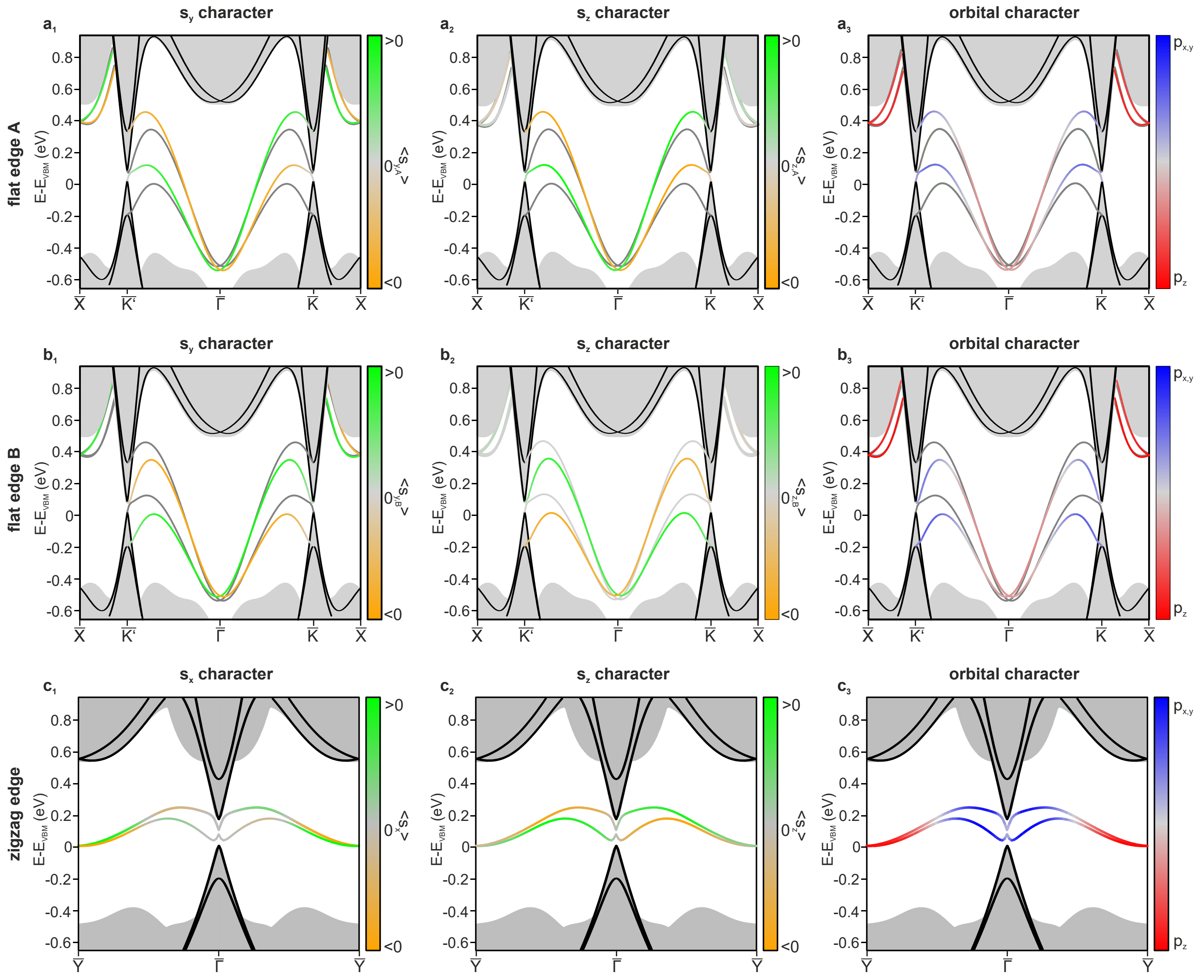

Figure S 4. Tight-binding slab calculations conducted for the flat edge **a,b** and zigzag edge geometry **c**, schematically illustrated in Fig. S3. The color code represents the spin and orbital character of each edge termination. For clarity, light gray indicates bulk bands of the slab, with a selected bulk dispersion overlaid in black. For the flat terminations **a,b**, bands from the opposite slab side are shown in darker gray. Note that the offset between edge and bulk bands at the Γ-point of zigzag edges is an artifact due to the finite slab geometry and vanishes in an infinite slab.

Decay constant calculations

The theoretical decay constant of edge states can be computed by calculating the local density of states (LDOS) from the TB Hamiltonian (1) and integrating over all in-gap states created by the n Kramers pairs. More precisely, the LDOS can be written as

$$\mathrm{LDOS}_{n,\boldsymbol{k}}^{i,m} = |c_{n,\boldsymbol{k}}^{i,m}|^2 \tag{9}$$

where $c_{n,\boldsymbol{k}}^{i,m}$ is the electron wavefunction coefficient at site i, orbital m, band n and wavevector $\boldsymbol{k}$ that diagonalizes H. To take the intrinsic sensitivity of STS to electronic states with out-of-plane character in to account [12], we further multiply the LDOS by the square modulus of the indium 5p hydrogen-like wavefunction $\phi_{nlm}\left(\boldsymbol{r}\right)$ with $n=5$, $l=1$ and $m=x,y,z$ to obtain the energy-dependent charge density profile $n\left(\omega,\boldsymbol{r}\right)$ accessible by STS, explicitly

$$n\left(\omega,\boldsymbol{r}\right) = \sum_{\boldsymbol{k},n}\sum_{i,m}\delta\left(\omega - E_{n\boldsymbol{k}}\right)|c_{n,\boldsymbol{k}}^{i,m}\cdot\phi_m\left(\boldsymbol{r}-\boldsymbol{R_i}\right)|^2 \tag{10}$$

where R_i is the lattice vector referred to the i-*th* In atom, $\delta(x)$ is the Dirac function, $E_{n\boldsymbol{k}}$ is the energy of an electron in the n-*th* band with wavevector $\boldsymbol{k}$ and ω is the bias. The decay constant is then computed by fitting the in-gap signal $n\left(\boldsymbol{r}\right)=\int_{\mathrm{gap}} n\left(\omega,\boldsymbol{r}\right)d\omega$ profiles with an exponential function $\sim e^{-x/\xi}$, as show in Fig. S3. We obtain $\xi=(3.7\pm0.3)$ Å and $\xi=(3.1\pm0.3)$ Å for the zigzag and flat edge A, respectively.

SUPPLEMENTARY DISCUSSION IV

As discussed in the main text, we attribute the backscattering free energy window within Δ_{sup} to the $n = 1$ Kramers pair interval of the flat edge band structure. This conclusion is based on the excellent agreement of the latter with our STS measurements as well as the finite limit Δ_∞ at large segments. To put it to a test, we examine the dynamical Coulomb blockade (DCB) scenario as the most plausible alternative mechanism for the spectral weight suppression near the Fermi level E_F and conclude that it is inconsistent with the observed metallic LDOS in Δ_{sup}, Δ_∞, as well as the behavior observed with changes in tunneling resistance.

The dynamical Coulomb blockade represents a charging effect in transport experiments that occurs when the applied bias voltage is smaller than the charging energy $E_C = 2e^2/C_\Sigma$ of the system. This is frequently the case in STS due to the relatively small capacitances involved in the tunneling experiment and thus often leads to a parasitic gap at the Fermi level in STS measurements [13].

In the dynamical Coulomb blockade framework, the tunneling experiment is modeled as a double junction as depicted in Fig. S5a. It consists i) of the tip-surface junction, characterized by R_T and C_T and ii) the surface-substrate junction that describes the surface coupling to the substrate through R and C. The tunneling resistance R_T is defined by the tunneling set point $R_\text{T} = V_\text{set}/I_\text{set}$ and typically ranges from $0.5\,\text{G}\Omega$ to $10\,\text{G}\Omega$ in our experiments. The associated tunneling capacitance C_T is usually $C_\text{T} < 1\,\text{aF}$ and depends on R_T, since R_T is also a measure of the distance between tip and sample surface that enters C_T. The parameter of the second ii) junction are usually adjusted in the DCB model assuming $R \ll R_\text{T}$, allowing the model to depend primarily on the two parameters R and $C_\Sigma = C_\text{T} + C$ [13, 14].

First, we provide insight into how R and C_Σ suppress the differential conductance $\text{d}I/\text{d}V(E)$ near E_F by modeling exemplary parameters in Fig. S5b,c. While R primarily affects the slope (Fig.5b), C_Σ determines the width of the DCB gap, as illustrated in Fig. S5b,c. We identify the regime $R > 10R_\text{K}$ and $\text{C}_\Sigma < 1\text{aF}$ for accurately reproducing the energy scale and hat-shape of Δ_{sup}, where $R_\text{K} = h/e^2 \approx 25.8\,\text{k}\Omega$ is the resistance quantum. Note that, at the energy scale of Δ_{sup}, the DCB model would inevitably produce a hard gap in STS, which is in clear contradiction with the metallic states observed within Δ_{sup}. Furthermore, the low C_Σ required for producing such a DCB gap, indicates that C and C_T are of comparable size, rendering the DCB gap sensitive to changes in C_T. In order to test Δ_{sup} for DCB-like behavior, we thus control C_T via the tunneling resistance R_T, specifically by tuning $V_\text{set}/I_\text{set}$.

Accordingly, we record $\text{d}I/\text{d}V(E,x)$ line scans along the indenene edge consisting of two flat edge A segments adjacent to hydrogen saturated SiC, as shown in Fig. 5d$_1$ and d$_2$. Both segments act as scattering resonators for the edge states, that exhibit the previously observed QPI E_i modes and QPI suppression within the Δ_{sup} (see Fig. S5b). Corresponding high-resolution zoom-in STS scans are shown in Fig. S5c and d. The $\text{d}I/\text{d}V(E,x)$ line scans were taken at $R_\text{T} = 10\,\text{G}\Omega$ (panel f) and $R_\text{T} = 0.8\,\text{G}\Omega$ (panel g), respectively, which translates to a difference of $\Delta z = -1.5$ Å in the tip to sample distance.

The change in tunneling resistance does not show any significant differences in the QPI onset within Δ_{sup} that would indicate the presence of a DCB gap, see red arrows in Fig. S5f$_2$ and g$_2$. This is even more apparent in the mean curves of both line scans shown in Fig. S5g$_1$, where the QPI onset within Δ_{sup} remains identical for both R_T values. Based on previous work [15], we estimate that reducing R_T from $R_\text{T} = 10\,\text{G}\Omega$ to $R_\text{T} = 0.8\,\text{G}\Omega$ increases C_T and thus C_Σ by at least $0.1\,\text{aF}$. Contrary to our observation, changing C_T on that scale would reduce the size of the hard gap within the DCB model, as indicated in Fig. S5c, where orange and purple arrows mark the onset of the hypothetical DCB gap.

Overall, we conclude that the LDOS suppression within Δ_{sup} is inconsistent with a DCB, because 1. the large segment length limit $\Delta_\infty = (0.26 \pm 0.06)$ eV is finite (Fig. 3f of the main text); 2. the shape and energy scale of Δ_{sup} would produce a hard gap in the DCB model contrary to the observed metallic LDOS and 3. variations in the tunneling resistance R_T and the associated tunneling capacitance C_T do not affect the QPI onset within Δ_{sup}, contrary to expectations from the DCB model.

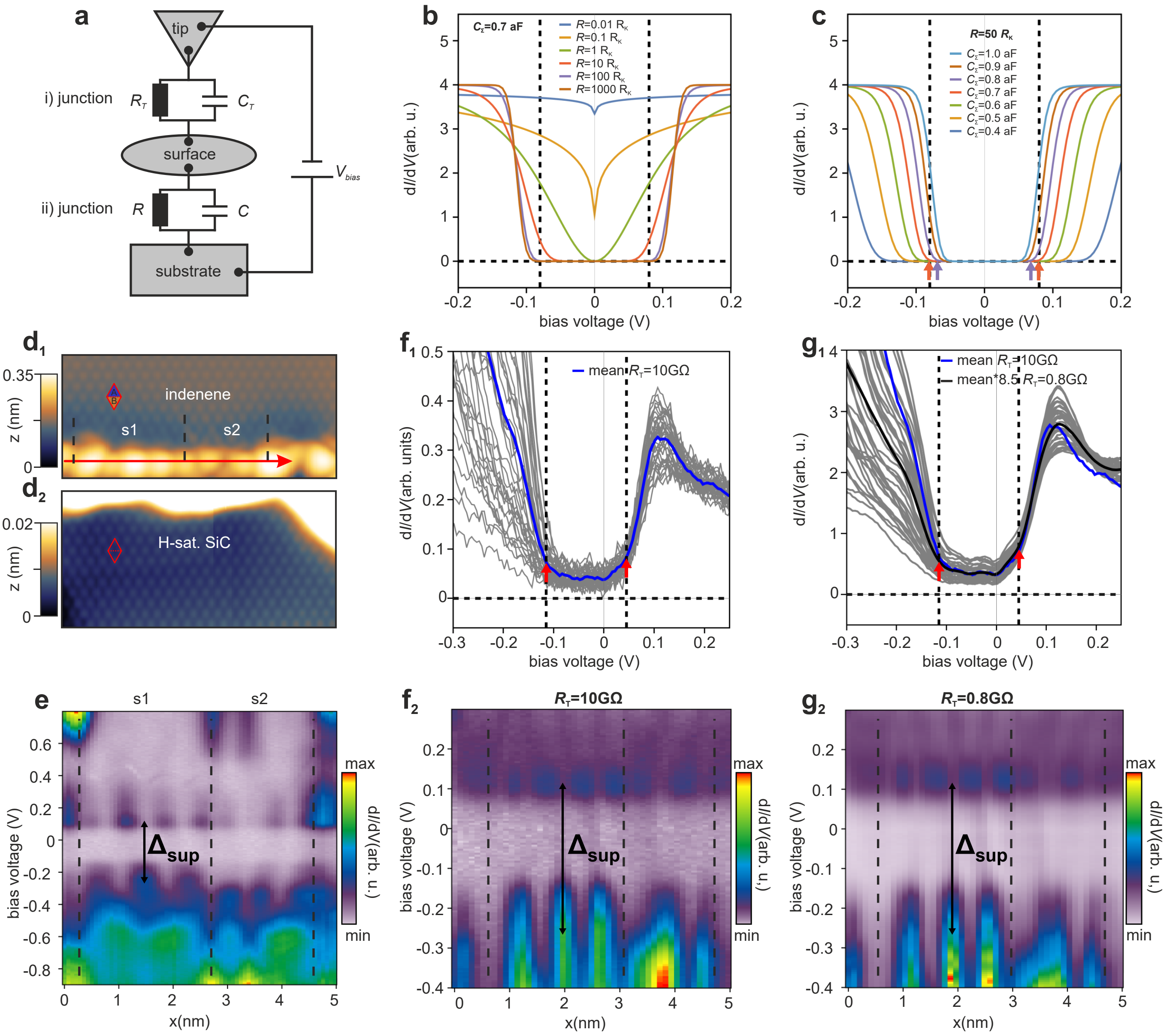

Figure S 5. **a** Schematic picture of electron tunneling through a double-junction comprising the i) tunnel junction characterized by R_T and C_T and the dissipative ii) junction characterized by R, C. **b** DCB model evaluated according to Refs. 13 and 14 for $C_\Sigma = 0.7\,\mathrm{aF}$ and various R. A DCB gap shape similar to experimentally observed $\mathrm{d}I/\mathrm{d}V(E)$ suppression within Δ_sup (indicaed by dashed lines) is achieved for $R > 10\,R_\mathrm{K}$. **c** DCB model evaluated for $R = 50\,R_\mathrm{K}$ and various C_Σ. Hard DCB gaps that suppress the $\mathrm{d}I/\mathrm{d}V(E)$-signal in a similar energy range to the experimental observations are generated for $C_\Sigma = (0.7 - 0.8)\,\mathrm{aF}$, as indicated by the arrows marking their respective onsets. $\mathbf{d}_1$ Atomically resolved STM scan of an indenene flat edge A to H-sat. SiC taken at $I_\mathrm{T} = 500\,\mathrm{pA}$, $\mathrm{V}_\mathrm{bias} = 0.6\,\mathrm{V}$. The indenene unit cell is shown as an inset. $\mathbf{d}_2$ Atomically resolved STM topography of H-sat. SiC(0001) taken at $I_\mathrm{T} = 50\,\mathrm{pA}$, $\mathrm{V}_\mathrm{bias} = 1.8\,\mathrm{V}$ and indicated SiC(0001) unit cell. **e** Line $\mathrm{d}I/\mathrm{d}V(V,x)$ scan taken along the red arrow in $\mathbf{d}_1$ with scan parameters $I_\mathrm{T} = 1000\,\mathrm{pA}$, $\mathrm{V}_\mathrm{bias} = -1\,\mathrm{V}$. **f** Line $\mathrm{d}I/\mathrm{d}V(V,x)$ scan taken at $R_\mathrm{T} = 10\,\mathrm{G\Omega}$ along the red arrow in $\mathbf{d}_1$ and scan parameters $I_\mathrm{T} = 50\,\mathrm{pA}$, $\mathrm{V}_\mathrm{bias} = 0.4\,\mathrm{V}$. $\mathbf{f}_1$ Collective plot of all $\mathrm{d}I/\mathrm{d}V(V)$ curves (gray) as well as their mean (blue) and $\mathbf{f}_2$ spatially resolved false color plot of $\mathrm{d}I/\mathrm{d}V(V,x)$. **g** Line $\mathrm{d}I/\mathrm{d}V(V,x)$ scan similarly taken to **f** with reduced tunneling resistance $R_\mathrm{T} = 0.8\,\mathrm{G\Omega}$ ($I_\mathrm{T} = 500\,\mathrm{pA}$, $\mathrm{V}_\mathrm{bias} = 0.4\,\mathrm{V}$). $\mathbf{g}_1$ Collective plot of all $\mathrm{d}I/\mathrm{d}V(V)$ curves (gray) and their mean (black) and $\mathbf{g}_2$ spatially resolved false color plot of $\mathrm{d}I/\mathrm{d}V(V,x)$. For comparison the $R_\mathrm{T} = 10\,\mathrm{G\Omega}$ mean $\mathrm{d}I/\mathrm{d}V(V)$ curve is overlaid and scaled up by a factor of 8.5.

SUPPLEMENTARY DISCUSSION V

In analogy to flat edge A discussed in the main text, we investigate band structure and QPI pattern of flat edge B. Our corresponding tight binding analysis is depicted in Fig. S6**a** with the colorcode indicating the $\langle s_y \rangle$ character and yields Rashba-like bands at the Brillouin zone center similar to those observed for flat edge A. However, when approaching the projected $\overline{K}$ and $\overline{K'}$ of the bulk bands the deviation from the flat edge A (gray in **a**) becomes evident in local band maxima and strong downwards bending of the flat edge B bands. These deviations can be attributed to the local edge potential at flat edge B, as its edge bands connect the second valence band to the first conduction band of the projected bulk band structure (black in Fig. S6**a**), thereby following the valence and conduction band splitting induced by the in-plane ISB term, which is in turn related to the edge potential arising from the carbon atom of the SiC surface (see Fig.2**a**).

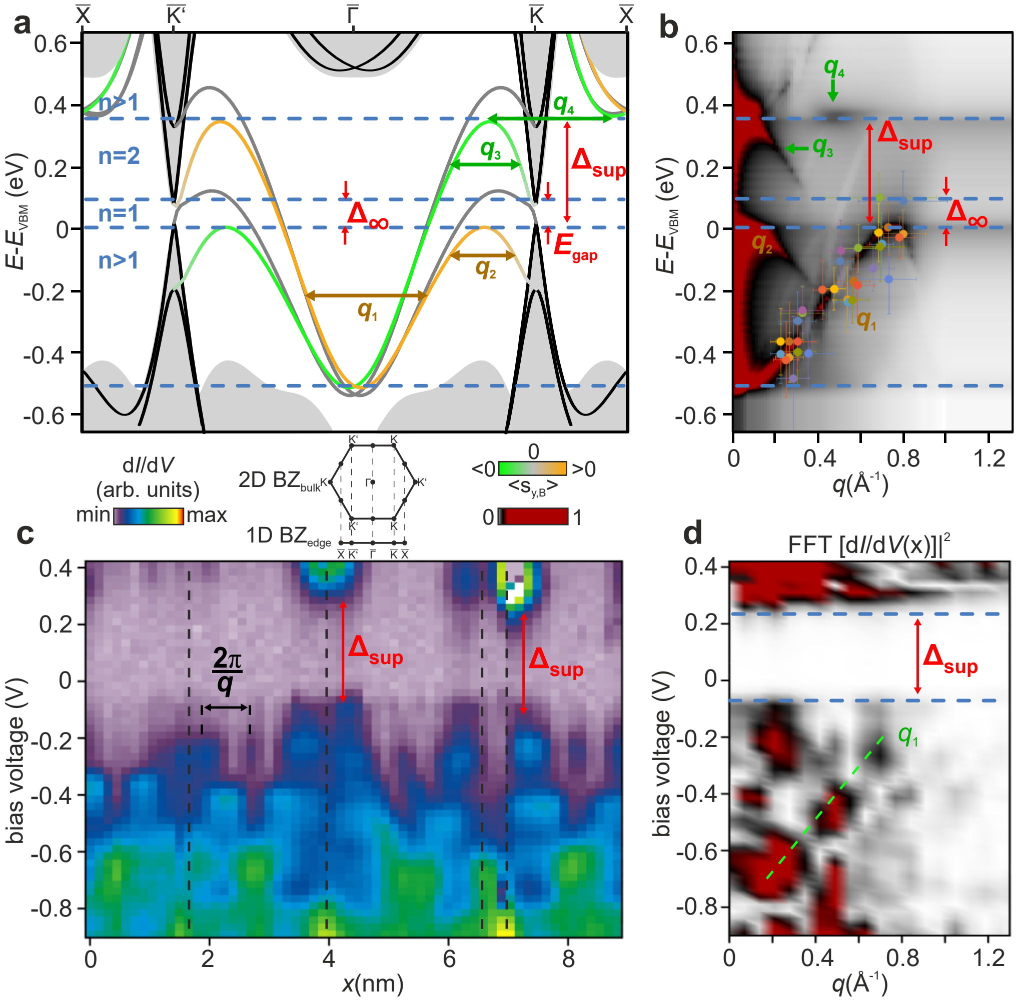

Figure S 6. **a** Tight binding slab calculations (gray) of indenene flat edges with flat edge B localized bands color-coded according to their $\langle s_y \rangle$ character. For clarity, selected bulk bands (black) indicate valence and conduction band onsets. Representative momentum transfers q_1-q_4 of TRS-allowed backscattering processes are illustrated by arrows. Δ_∞ marks an $n = 1$ Kramers pair interval and Δ_{sup} the separation of parabolic edge bands centered at $\overline{X}$ and the local edge band maxima between $\overline{\Gamma}$ and $\overline{K}$. **b** Flat edge B energy-dependent JDOS $E(q)$ of q involved in scattering processes that are facilitated by the dispersion of flat edge calculated according to Supplementary Discussion III. For clarity, four scattering branches q_1-q_4 are distinguished.Data points are extracted from experimentally observed resonator oscillations (12 flat edge B segments represented by different colors) are plotted as an overlay offset to compensate for the experimental n-doping, using the valence band maximum (VBM) as a reference point, see Method section. **c** Line $\mathrm{d}I/\mathrm{d}V(E,x)$ scan of a indenene flat edge A taken with tunneling setpoint $I_{\text{T}} = 500\,\text{pA}$, $V_{\text{bias}} = -0.9\,\text{V}$. Segments separated by defects are indicated by dashed lines and show QPI-related bias dependent $\mathrm{d}I/\mathrm{d}V$ peaks. **d** Squared modulus of the Fourier transformed line scan in panel **c** showing q_1 related spectral weight (dashed green line) and QPI suppression within Δ_{sup}.

These changes to the band dispersion significantly affect the available momentum transfers summarized in branches q_1 to q_4 of the calculated momentum JDOS depicted in Fig. S6**b**. Specifically, the maximal $q_{\mathrm{B,max}} \approx 0.8\text{Å}^{-1}$ is shorter than for flat edge A ($q_{\mathrm{A,max}} \approx 1\text{Å}^{-1}$), an observation we confirm by analyzing $2\pi/q$ from real space modulations in 12 flat edge B segments that are overlaid to the calculated QPI pattern in Fig. S6**b**. The corresponding real-space $\mathrm{d}I/\mathrm{d}V$ modulation is exemplified in the line $\mathrm{d}I/\mathrm{d}V$ scan shown in Fig. S6**c**, with its Fourier transform in Fig. S6**e** showing reasonable agreement with the occupied part of the calculated momentum JDOS.

As an additional modification to the band dispersion, the $n = 1$ Kramers pair interval Δ_∞ reduces to approximately 100 meV. However, due to the limited lengths of available edge segments, it is important to note for the experimental determination of Δ_∞ that, unlike flat edge A, its upper onset is defined by a relatively short q-wavevector (as shown in Fig. S6**b**), which decreases further at higher energies due to its hole-like character. In other words, the available edge segment lengths are insufficient to support standing waves associated with the scattering process that defines the upper onset of Δ_∞, making it impossible to extract a reliable value for flat edge B under the given constraints. The larger $\mathrm{d}I/\mathrm{d}V$ suppression region Δ_{sup} observed in the flat edge B segments (Fig. S6**c**) instead overestimates Δ_∞. Rather than corresponding to the true upper onset of Δ_∞, the features at $\sim 0.3\,\mathrm{V}$ align with scattering from the S-shaped band maximum to the X-point-centered parabola (in Fig. S6**a**), producing larger q-values that are compatible with the available segment lengths.

Overall, the observed standing wave pattern at flat edge B aligns well with the momentum JDOS derived from tight binding slab calculations. However, a definitive investigation of Δ_∞ requires longer flat edge B segments capable of supporting the shorter q-vectors in the particle-in-a-box spectrum.

SUPPLEMENTARY DISCUSSION VI

In the main text, we primarily focus on flat edges due to their band dispersion relation, which enables larger momentum transfers q in backscattering processes. This results in distinct standing waves within experimentally accessible edge lengths of up to 4 nm. In contrast, the zigzag edge has a smaller 1D Brillouin zone, resulting in shorter allowed momentum transfers that require longer edge segments on the 10 nm scale for demonstration. Given these constraints, we show that our observations at the shorter zigzag edge still align with its band structure and the corresponding backscattering processes.

We begin with a topography scan of the indenene boundary to H-saturated SiC (Fig. S7a), revealing the characteristic $\times\sqrt{3}$ periodicity of the zigzag edge, which arises from the diagonal termination of the indenene bulk unit cell. The same $\times\sqrt{3}$ periodicity is observed in the line STS scans along this edge segment shown in Fig. S7b, at both high ($\approx 0.8\,\mathrm{V}$) and lower bias voltages (-0.5 V). These regions are separated by an energy window of relatively low $\mathrm{d}I/\mathrm{d}V(E,x)$ signal, that reveals metallic states upon closer investigation at lower tunnel resistances, as demonstrated in Fig. S7c. The $\mathrm{d}I/\mathrm{d}V(E,x)$ signal is only weakly modulated along the zigzag segment, indicating that the scattering processes involved employ rather short momentum transfers.

To gain insight into these processes, we conduct tight binding slab calculations (see Supplementary Discussion III) of the zigzag edge termination, with results depicted in Fig. S7d. Unlike the flat edges, this edge geometry projects the indenene bulk K-point (see black bands) to the Γ-point of the smaller 1D Brillouin zone. The zigzag edge bands completely fill the 1D Brillouin zone and shift the Kramers degenerate crossing to the Y-point, contrary to, e.g., bismuthene, where edge bands cross at the Γ-point [16]. We note that the offset between edge and bulk bands at the Γ-point is an artifact due to the finite slab geometry and vanishes in an infinite slab.

This mustache shaped dispersion closely resembles that of the Bi(111) armchair edge and is likely promoted by the multiorbital p_y and p_z character of these indenene edge states [7, 17]. It can be subdivided into energy sections with $n = 2$ and $n = 3$ Kramers pairs, the latter fully spanning the indenene bulk band gap and thereby enabling elastic single particle backscattering within this gap. For both regions, we employ the T-matrix formalism described in section to calculate the momentum JDOS $E(q)$ relation of momentum transfers q associated with spin-conserving backscattering processes. The results are shown in Fig. S7e and reveal that most scattering processes, such as $q_{1,2}$, involve short momentum transfers, whose corresponding wavelength $2\pi/q$ exceeds the length of typical zigzag segments. For the zigzag segment discussed in Fig. S7a-c, no scattering processes below $3\,\mathrm{nm}^{-1}$ (red line in panel e) satisfy the resonator condition set by the segment length of $L \approx 2$ nm. Scattering by the momenta $q_{1,2}$ is thus only feasible for longer edge segments, leaving only the Umklapp-like q_3 process. However, also the associated q_3 E_{-1} level produces only a single oscillation at this segment length, rendering extraction of $2\pi/q$ cumbersome and unconvincing. The same applies to the Fourier transform of Fig. S7c shown in f, where no periodicity beyond $0.3\,\mathrm{Å}^{-1}$ is observed. We conclude that scrutiny of the zigzag edge dispersion through backscattering inevitably requires longer edge segments, the development and investigation of which we will leave for future studies.

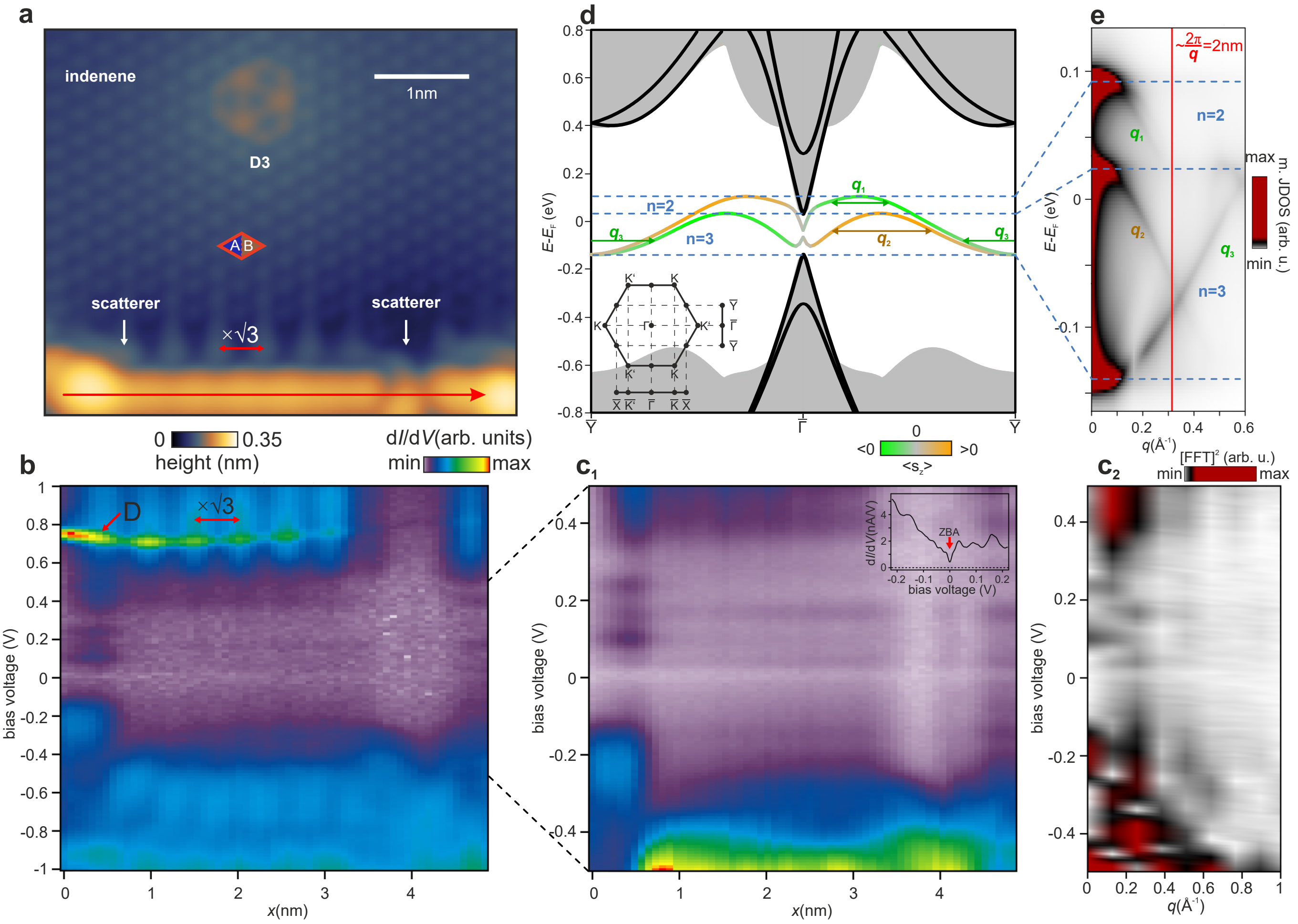

Figure S 7. **a** STM topography of the indenene boundary to H-sat. SiC taken at $I_\mathrm{T} = 50\,\mathrm{pA}$, $V_\mathrm{bias} = 0.5\,\mathrm{V}$. The long red arrow indicates the path of the $\mathrm{d}I/\mathrm{d}V(V, x)$ scan shown in **b**,**c**$_1$. White arrows mark shifts in the edge registry defining the length L of the zigzag segment. For clarity, an indenene bulk unit cell is shown as an inset. **b**,**c**$_1$ Line $\mathrm{d}I/\mathrm{d}V(V, x)$ scan taken along the red arrow in **a** with scan parameters $I_\mathrm{T} = 250\,\mathrm{pA}$, $V_\mathrm{bias} = -1\,\mathrm{V}$, $V_\mathrm{LockIn,rms} = 15\,\mathrm{mV}$, (**b**) and $I_\mathrm{T} = 250\,\mathrm{pA}$, $V_\mathrm{bias} = -0.5\,\mathrm{V}$, $V_\mathrm{LockIn,rms} = 15\,\mathrm{mV}$ (**c**$_1$). The sharp and intense $\mathrm{d}I/\mathrm{d}V(V)$ signal labeled D in **b** is attributed to an SiC donor energy level, see Supplementary Information of Ref. 20. The inset in **c**$_1$ shows a metallic spectrum of this edge including a zero bias anomaly (ZBA). **c**$_2$ Fourier transform of **c**$_1$ employing a Hann windowing function. **d** Tight binding slab calculations of indenene zigzag edge showing the $\langle s_z \rangle$ character of the zigzag edge bands, as indicated by the color code. Energy regions are separated by blue dashed lines based on the number of Kramers pairs n. Arrows indicate three distinct scattering channel associated with the momenta q_1, q_2 and q_3 at selected energies. **e** Corresponding energy-dependent momentum JDOS $E(q)$ of the momenta q_i calculated using the formalism of Supplementary Discussion III. A red line indicates the minimal q that satisfies the resonator condition for the edge segment shown in **a**.

SUPPLEMENTARY DISCUSSION VII

In this section, we outline the standard procedure for performing edge scanning tunneling spectroscopy (STS) measurements at the example of extended data on the flat edge A. Beginning with the overview scan shown in Fig. S8a, we identify straight boundaries (marked in red) between indenene and hydrogen-saturated (H-sat) SiC. The H-sat. regions manifest as holes in the otherwise continuous indenene film and are readily distinguishable in STS, where the 3.2 eV band gap of SiC serves as an unambigeous fingerprint, as demonstrated by the associated $\mathrm{d}I/\mathrm{d}V$ spectrum depicted as an inset. The absence of SiC states within this wide energy window allows us to exclude hybridization between the indenene edge bands and the substrate. Before approaching the edge itself, we first calibrate the lattice orientation as described in Supplementary Discussion I, utilizing nitrogen dopants that substitute carbon in SiC and thus indicate site B of the indenene unit cell. By comparing the defect orientation shown in Fig. $8b_1$ with the edge in Fig. $S8b_{2,3}$, we can confidently identify the latter as A-terminated indenene. Note that the atomically resolved topography scan reveals a pronounced edge corrugation (Fig. $S8b_2$), which is ascribed to the electronic QPI pattern and intermixes with the otherwise well defined flat edge. Due to this admixture of LDOS contributions in topography scans, we optimize the stabilization bias voltage for edge line STS scans to have the lowest possible edge corrugation, a condition reliably achieved at low negative bias voltages as shown in Fig. $S8b_3$.

On the opposite side of the indenene edge, we identify the SiC lattice within the 1×1 surface unit cell, which can only be stabilized in the hydrogen-saturated form (Fig. $S8b_4$) [18]. It is important to note that the large band gap of SiC necessitates higher bias voltages for atomically resolved STM imaging of the SiC surface, thereby prohibiting simultaneous imaging of both the indenene and H-sat. SiC surfaces.

Having identified the A-termination of this flat edge, we are now ready to examine its spectroscopic features. Fig. 8a presents the corresponding 10 nm $\mathrm{d}I/\mathrm{d}V(E,x)$ line scan, following the red path along the indenene edge topography, as indicated in Fig. $8b_3$. Based on the topographic features, we divide the linescan into four segments (1 to 4), separated by kinks in the edge (dashed lines) that act as scattering centers, thereby defining the quantum resonators observed in Fig. S8c. As indicated in segment 1, the QPI levels are suppressed between E_{-1} and E_{+1} within Δ_{sup}. As outlined in the main text, quantitative variations of Δ_{sup} among different segments of this line scan, are attributed to the resonator condition as prescribed by the segment length L.

The wavelength $2\pi/q$ of the standing wave $\mathrm{d}I/\mathrm{d}V(E,x)$ modulation depends on the scattering-related momentum transfer q, and thus gives access to the scattering processes supported by the edge band dispersion. Due to the relatively short straight edge segments, we analyze $2\pi/q$ directly in real space, in analogy to what is presented in Ref. 19. This method is exemplary shown in Fig. $S8c_1$ to c_6 for segment 4, depicting line profiles $\mathrm{d}I/\mathrm{d}V(x)$ for bias voltages related to energy levels E_{-1} to E_{-6}. Each maxima (red dots) is fitted by a Gaussian marked by dashed red line whose center is indicated by a red arrow. $2\pi/q$ is then calculated as the difference of adjacent maxima. Its error bar contains the standard error of multiple $2\pi/q$ values at a single bias voltages V as well as the width of the Gaussian. The error in V corresponds to the width of the $\mathrm{d}I/\mathrm{d}V(E,x)$ peaks on the bias axis. In contrast, for the lowest level of each segment, which is E_{-7} in segment 4, $2\pi/q$ has to be estimated from the resonator length (dashed black lines in panel c) and is therefore less accurate. E_{-7} is already evident as the central peak in panel c_1, indicating that the level spacing diminishes at these lower energies, consistent with the calculated non-linear dispersion relation of Fig. 4 in the main text. This analysis straightforwardly yields the $E(q)$ dependence for each flat edge segment that can be compared to the calculated momentum JDOS $E(q)$ relation in Fig. 4b,d of the main text.

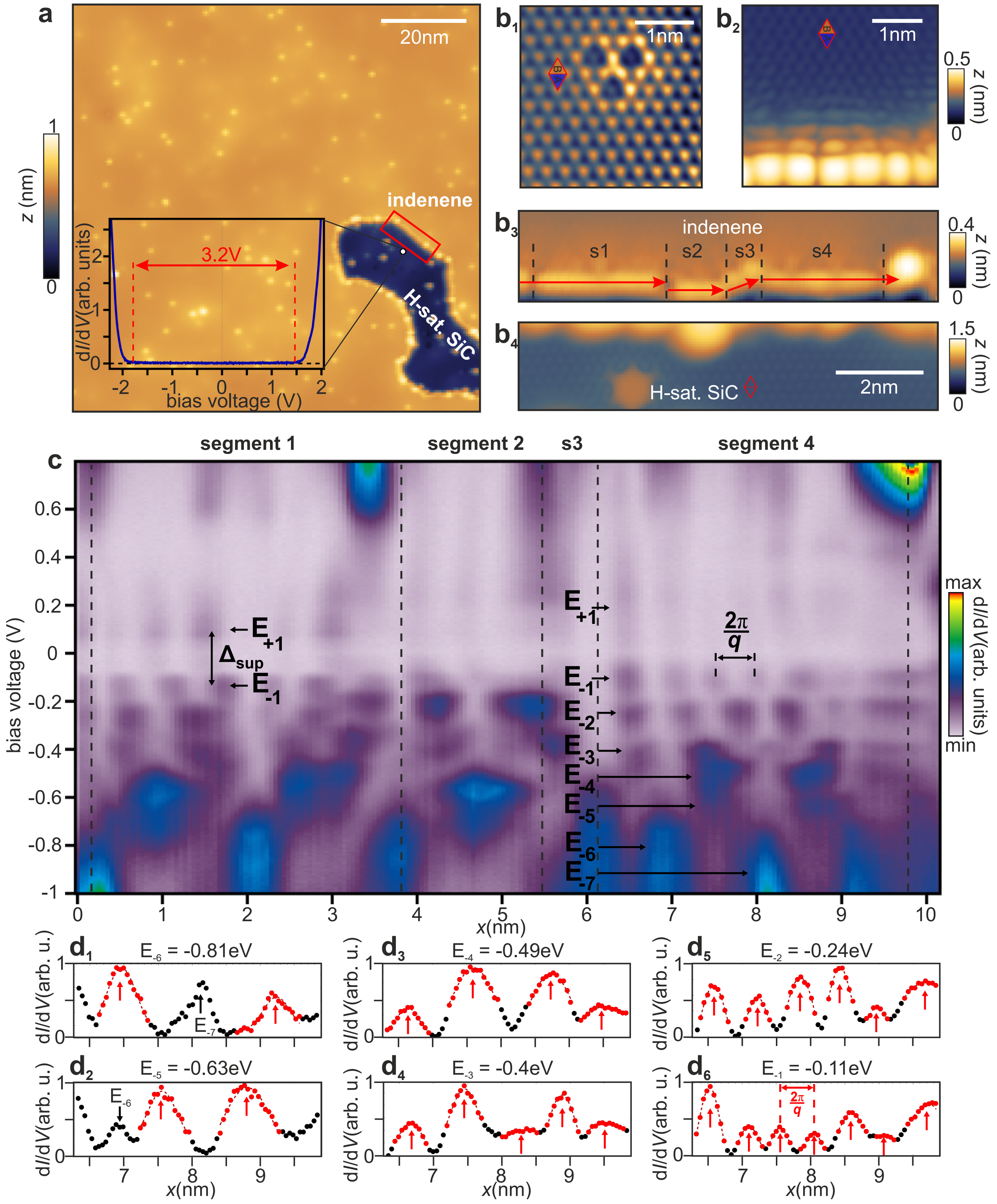

Figure S 8. **a** STM overviewscan showing in red a straight interface between an hole with H-sat. SiC in the otherwise closed indenene film, showing a SiC STS spectrum with the $\sim 3.2\,$eV gap of 4H-SiC [21] as an inset (topography: $I_T = 10\,$pA, $V_{bias} = 3\,$V, inset: $I_T = 50\,$pA, $V_{bias} = 1.8\,$V). **b**$_1$ Subsurface D1 N-defect pinpointing the B-site within the bulk indenene unit cell, from which the A-termination of the edge (**b**$_2$) is derived, see Supplementary Discussion I. **b**$_{3,4}$ Edge STM topography of the region marked in **a**, showing the featureless indenene film at $I_T = 500\,$pA, $V_{bias} = -1\,$V (**b**$_3$) and the atomically resolved H-sat. SiC(0001) surface at $I_T = 50\,$pA, $V_{bias} = 1.8\,$V (**b**$_4$). **c** Line $dI/dV(E, x)$ scan taken along the path indicated in **b**$_3$ with setpoint parameters $I_T = 750\,$pA, $V_{bias} = -1\,$V, $V_{mod,rms} = 10\,$mV. Dashed lines indicate segments 1 to 4 (also in **b**$_3$) separated by kinks in the indenene edge topography. The insets exemplarily mark the energy levels E_i as well as the separation $2\pi/q$ of the resonator modes. **d**$_1$ to **d**$_6$ Line $dI/dV(x)$ profiles illustrate the analysis of the spatial mode separation $2\pi/q$ for bias voltages related to E_{-1} to E_{-6} of segment 4. Sections of red dots are fitted by a Gaussian function (dashed curve) whose center is indicated by red arrows.

[1] Bockstedte, M., Mattausch, A. & Pankratov, O. Solubility of nitrogen and phosphorus in 4H-SiC: A theoretical study. *Appl. Phys. Lett.* **85**, 58–60 (2004).

[2] Ferro, G. & Chaussende, D. A new model for in situ nitrogen incorporation into 4H-SiC during epitaxy. *Sci. Rep.* **7**, 43069 (2017).

[3] Weidlich, P. H., Dunin-Borkowski, R. E. & Ebert, P. Quantitative determination of local potential values in inhomogeneously doped semiconductors by scanning tunneling microscopy. *Phys. Rev. B* **84**, 085210 (2011).

[4] Ferreira da Silva, A. *et al.* Electrical resistivity and metal-nonmetal transition in n-type doped $4H-$SiC. *Phys. Rev. B* **74**, 245201 (2006).

[5] Bauernfeind, M. *et al.* Design and realization of topological Dirac fermions on a triangular lattice. *Nat. Commun.* **12**, 5396 (2021).

[6] Schmitt, C. *et al.* Achieving environmental stability in an atomically thin quantum spin Hall insulator via graphene intercalation. *Nat. Commun.* **15**, 1486 (2024).

[7] Eck, P. *et al.* Real-space obstruction in quantum spin Hall insulators. *Phys. Rev. B* **106**, 195143 (2022).

[8] Moldovan, D. & Peeters, F. pybinding v0.8.0: a python package for tight- binding calculations (2016).

[9] Kohsaka, Y. *et al.* Spin-orbit scattering visualized in quasiparticle interference. *Phys. Rev. B* **95**, 115307 (2017).

[10] Tresca, C. *et al.* Chiral spin texture in the charge-density-wave phase of the correlated metallic Pb/Si(111) monolayer. *Phys. Rev. Lett.* **120**, 196402 (2018).

[11] Deretzis, I., Calogero, G., Angilella, G. G. N. & La Magna, A. Role of basis sets on the unfolding of supercell band structures: From tight-binding to density functional theory. *EPL* **107**, 27006 (2014).

[12] Wiesendanger, R. *Scanning Probe Microscopy and Spectroscopy Methods and Applications* (Cambridge University Press, UK-CB2 2RU Cambridge, 1998).

[13] Brun, C. *et al.* Dynamical Coulomb Blockade Observed in Nanosized Electrical Contacts. *Phys. Rev. Lett.* **108**, 126802 (2012).

[14] Joyez, P. & Esteve, D. Single-electron tunneling at high temperature. *Phys. Rev. B* **56**, 1848–1853 (1997).

[15] van Bentum, P. J. M., van Kempen, H., van de Leemput, L. E. C. & Teunissen, P. A. A. Single-electron tunneling observed with point-contact tunnel junctions. *Phys. Rev. Lett.* **60**, 369–372 (1988).

[16] Reis, F. *et al.* Bismuthene on a SiC substrate: A candidate for a high-temperature quantum spin Hall material. *Science* **357**, 287–290 (2017).

[17] Wang, Z. F., Chen, L. & Liu, F. Tuning Topological Edge States of Bi(111) Bilayer Film by Edge Adsorption. *Nano Lett.* **14**, 2879–2883 (2014).

[18] Glass, S. *et al.* Atomic-Scale Mapping of Layer-by-Layer Hydrogen Etching and Passivation of SiC(0001) Substrates. *J. Phys. Chem. C* **120**, 10361–10367 (2016).

[19] Stühler, R. *et al.* Effective lifting of the topological protection of quantum spin Hall edge states by edge coupling. *Nat. Commun.* **13**, 3480 (2022).

[20] Syperek, M. *et al.* Observation of room temperature excitons in an atomically thin topological insulator. *Nat. Commun.* **13**, 6313 (2022).

[21] Seyller, T. Electronic properties of sic surfaces and interfaces: some fundamental and technological aspects. *Applied Physics A* **85**, 371–385 (2006).
